# Calibration of Low-Cost Capacitive Soil Moisture Sensors for Irrigation Management Applications

**DOI:** 10.3390/s25020343

**Published:** 2025-01-09

**Authors:** Ahmed A. Abdelmoneim, Christa M. Al Kalaany, Roula Khadra, Bilal Derardja, Giovanna Dragonetti

**Affiliations:** Sustainable Water and Land Management in Agriculture, The Mediterranean Agronomic Institute (CIHEAM Bari), 70010 Valenzano, Bari, Italy; christamariakly@gmail.com (C.M.A.K.); derardja@iamb.it (B.D.); dragonetti@iamb.it (G.D.)

**Keywords:** capacitive sensors, SEN0193, soil moisture, precision agriculture, microcontroller, ESP32

## Abstract

The calibration of capacitive soil moisture sensors is an essential step towards their integration into smart solutions. This study investigates the calibration of a widely used low-cost capacitive soil moisture sensor (SKU:SEN0193, DFRobot, Shanghai, China) in a loamy silt soil typically found in the Puglia region of Italy. The calibration function was derived from a random sample of 12 sensors, with three soil sample replicas per sensor, each of which had one of five gravimetric soil moisture contents, from relatively dry (5%) to full saturation (40%). The study reports the resulting calibration function along with the accuracy achieved with the generalized calibration function. The sensors proved to be accurate, with an R^2^ value ranging between 0.85 and 0.87 and a root mean square value (RMSE) ranging between 4.5 and 4.9%. The variation between the sensors was also investigated. The results showed that with higher soil moisture contents (above 30%), the sensor-to-sensor variability becomes significant, with a coefficient of variation (CV) ranging between 10 and 16%; meanwhile, in lower soil moisture contents, the CV ranged between 6.5 and 10.3%, implying that it is more consistent in lower moisture content within this soil condition. The resulting calibration function enhances the integration of such low-cost sensors into smart farming solutions. With proper calibration, these affordable capacitive sensors can achieve a high degree of accuracy, making them a viable option for widespread use in cost-effective precision agricultural applications.

## 1. Introduction

One of the major challenges facing humanity today is the increasing scarcity of water resources. Although water covers 71% of our planet, only 2.5% is fresh water, out of which only 1% is accessible [1]. Globally, irrigated agriculture represents 70% of the consumption of that freshwater [2,3]. Thus, efforts towards enhancing on-farm irrigation management are crucial to face such challenges for a finite resource [4].

Soil moisture monitoring is essential for enhanced on-farm irrigation management. Accurate data on soil moisture levels enable farmers to optimize irrigation practices, ensuring that crops receive the right amount of water at the right time [5,6]. One of the main challenges hindering the expansion of such enhancement is the lack of cost-effective and reliable data monitoring systems [4,7]. In this study, a low-cost capacitive soil moisture sensor will be calibrated, and its consistency will be investigated to evaluate its potential integration into an Internet of Things (IoT) soil moisture monitoring system.

Moisture conditions in soil are described using the following two terms: (i) mass/soil water content (SWC) and (ii) energy/soil water potential (SWP). This study will focus on SWC, as it investigates the reliability of physical capacitive soil moisture sensing. Nevertheless, SWP is essential to clearly understand soil moisture conditions [8,9,10].

Soil water content (SWC) can be defined as the volume (or weight) of the moisture existing in a certain soil sample in relation to the total volume (or weight) of the sample [4].

There are two main methods used to measure SWC: (i) direct methods and (ii) indirect methods [11,12]. Direct methods involve measuring the actual soil moisture gravimetrically by taking a soil sample and drying it in a lab oven at 105 °C for 24 h (the period and drying temperature may vary depending on the organic matter content of the soil). This method is labor-intensive, destructive, and time-consuming, but it is highly accurate [13,14,15]; thus, it is considered the standard when calibrating other methods [16]. Nevertheless, it is not suitable for in situ measurements.

Meanwhile, indirect methods involve measuring the variation in an internal property of the sensory system as an indirect indicator for soil water content variation [17]. These methods provide instantaneous, continuous, non-destructive measurements with acceptable accuracy [16,18,19,20]. Examples of indirect methods include the following: dielectric methods, neutron scattering, electrical resistance, electromagnetic sensing, soil electrical conductivity, the hygrometric method, and gamma ray attenuation. More in-depth information about each indirect method can be found in refs. [21,22].

Dielectric methods, which include time domain reflectometry (TDR), frequency domain reflectometry (FDR), and capacitance sensing, are the most used in situ techniques, due to their automation capability, relatively high accuracy, and easy installation [23,24,25,26,27].

Out of the three dielectric methods mentioned, capacitance sensors are often preferred for integration into smart farming systems due to their practicality and low cost compared to TDR and FDR [28,29,30,31]. However, the main disadvantage of the capacitive sensing technique is that it necessitates careful calibration due to the variations in the physical, chemical, and biological characteristics of soils and their impact on its electromagnetic characteristics [32,33,34]. Thus, many studies published in the literature have focused on capacitive soil moisture calibration for certain soils.

Components from the low-cost electronics market often come with limited technical documentation, requiring thorough evaluation beforehand to verify their functionality and specifications [35]. One of the most commonly used low-cost capacitive soil moisture sensors in commercial IoT nodes and for smart farming applications is the SKU:SEN0193 (DFROBOT, Shanghai, China). Its popularity is mainly driven by its low cost, the fact that it can easily be found in markets, and the fact that it is made of corrosion-resistant material, which increases its durability [34].

The SEN0193 costs around USD 8–10/unit; its affordability makes it an attractive option for applications in precision agriculture, particularly when scaling up to multiple sensors for large fields. Although other commercial sensors can feature advanced telemetry and full package solutions, such as SKU: TEROS 12 (METER group, Washington, D.C., USA), their relatively high cost (USD 200–250/unit) makes them mainly suitable for research or high-budget operations.

Recently, a number of studies were conducted on the calibration and validation of the SEN0193 using certain soils. Ref. [21] assessed the repeatability and reproducibility of capacitive soil moisture sensors across clay loam, sandy loam, and silt loam soil textures using mean range methods. Calibration was performed by comparing sensor data with soil moisture, measured via the thermogravimetric method. The findings revealed that sensor responses varied significantly with soil texture, necessitating texture-specific calibration. A polynomial calibration function (R^2^ ≥ 0.89) was determined to be the most suitable for accurately modeling sensor behavior across different soil types.

Using the same sensor, a similar experiment was conducted by [36] to calibrate the SEN0193 using the gravimetric method. The RMSE was 0.09 cm^3^ cm^−3^ for samples in the dry-to-saturated range and 0.05 cm^3^ cm^−3^ for samples in the field capacity range. The study concluded on the importance of the developed calibration function to use the SEN0193 sensor for water stress management.

Majumder et al. [37] evaluated the accuracy of the same sensor against time domain reflectometry (TDR). The results indicate that these sensors are highly accurate, exhibit low error rates (lower than 5%), and are a cost-effective alternative to TDR sensors, making them well-suited for large-scale deployments in agricultural and environmental applications.

Under silty clay soil, [38] calibrated the same sensor using the thermogravimetric method. The curve obtained had an adjusted R^2^ of 0.86 and a residual standard error of 2.8%. The study concluded that this sensor could be used for measuring the changes in soil moisture—within the error ranges—during rainy seasons or irrigation events.

Pereira et al. [39] calibrated the same sensor under three different soil types: Red-yellow Latosol (RYL), Regolitic Neosol (RN), and Red Latosol (RL). The resulting calibration function was highly correlated with the water content in the samples, with an R^2^ ranging from 0.93 to 0.96 for RYL, 0.89 to 0.92 for RN, and 0.86 to 0.88 for RL, while the RMSE values were 0.08, 0.12, and 0.15 cm^3^ cm^−3^ for RYL, RN, and RL, respectively.

Ref. [40] designed a solar-powered capacitive soil moisture sensor and calibrated it for clay soils. The calibration results demonstrated a strong correlation between sensor readings and estimated soil volumetric water content. Higher soil moisture content was associated with a decreased sensor output voltage, with an average determination coefficient (R^2^) of 0.967 and a root-mean-square error (RMSE) of 0.014.

On the other hand, Domínguez-Niño et al. [41] reported a significant variability when using capacitive soil moisture sensors. The study examined the uncertainties in the measurement process, along with the natural variability in the actual soil water dynamics. Measurements were collected from 57 sensors located at 10 combinations of depth and position relative to the drippers. The results showed large sensor-to-sensor differences, even when installed at equivalent depths and coordinates relative to the drippers. In contrast, differences among virtual sensors simulated using a HYDRUS-3D model at those soil locations were one order of magnitude smaller.

Rende and Biage [42] evaluated the performance of capacitive soil moisture sensors in irrigated soils, comparing them to traditional methods such as gravimetric measurements. The study highlighted the potential for capacitive sensors to improve water use efficiency by 15–20% when integrated into automated irrigation systems, making them a valuable tool for precision irrigation.

Fares and Alva [43] assessed the effectiveness of capacitance probes for soil moisture monitoring in citrus orchards. The key findings concluded that irrigation based on probe data reduced water consumption by 18–22% compared to conventional irrigation methods, which typically rely on fixed schedules. The probes helped in maintaining soil moisture at optimal levels (measured between 10–20% volumetric water content) for citrus root zones, which resulted in improved growth and increased yields by 12–15% over the growing season.

The performance of a capacitive soil moisture sensor was evaluated by [44] for monitoring the soil’s moisture content and its potential applications in irrigation management. The capacitive sensor effectively measured moisture within a range of 5–50% the volumetric water content (VWC) and was shown to improve irrigation efficiency by 15–20% when integrated into automated irrigation systems. Over a 6-month period, the sensor exhibited stable performance, although regular recalibration every 3–4 months was necessary to maintain its accuracy. These findings demonstrate the sensor’s potential as a reliable tool for real-time soil moisture monitoring, enhancing water use efficiency in agricultural irrigation practices.

In Mississippi, [45] evaluated and calibrated three soil moisture sensors (TDR315, Acclima, Meridian, ID, USA), CS655, Campbell Scientific Inc., Logan, UT, USA) and (GS1, METER Group Inc., Pullman, WA, USA) across six soil types, including clayey and loamy soils, using field and laboratory methods. Results showed that factory-calibrated sensors often overestimated soil volumetric water content, particularly in clayey soils, while performance was closer to gravimetric measurements in sandy loam. The RMSE ranged from 0.03 to 0.29 cm^3^/cm^−3^, with TDR315 performing better overall. Soil-specific calibration significantly improved accuracy, with field validation showing acceptable accuracy (R^2^ > 0.6) for TDR315 and CS655 when calibrated for undisturbed soil conditions.

In the majority of the previous studies, low-cost microcontrollers (MCUs) were used to convert the output voltage of the SEN0193 sensor into an analog output. An extensive literature review of prototyping of sensing systems for irrigation in precision agriculture was conducted by [46]. Of the 160 studies reviewed, 80% were published within the last 3 years. The findings also highlighted that the Arduino Uno microcontroller was the preferred prototyping MCU, particularly for applications that do not involve wireless sensor networks (WSNs).

In this study, an ESP32 Lolin V1.0 (Espressif systems, Shanghai, China) was used. The ESP32 Lolin is versatile, affordable, and suitable for IoT-based applications. It is equipped with a dual-core processor operating at up to 240 MHz, with 520 KB of SRAM and integrated Wi-Fi and Bluetooth capabilities, making it ideal for real-time data acquisition and wireless transmission. It features multiple general input/output pins and a 12-bit ADC, enabling precise analog-to-digital conversion, which is crucial for interfacing with soil moisture sensors. Additionally, its compact size and low power consumption make it suitable for field applications and integration into smart agriculture systems.

The decision to use the ESP32 Lolin was driven by its extensive community support, robust firmware ecosystem, and compatibility with widely used development environments such as Arduino IDE V2.3.4. and MicroPython, ensuring ease of programming and rapid prototyping.

To summarize the conclusions from the reviewed literature:

1. SKU:SEN0193 is a cost-effective sensor commonly used and distinguished by its practicality to integrate in IoT solutions,
2. However, it necessitates careful calibration with local conditions to be able to generate reliable results and to be integrated with smart farming systems.

Thus, the objectives of this study were to (i) calibrate the SEN0193 for soil typical of the Puglia (Italy) region (i.e., silty loam soil) against the thermogravimetric method, (ii) report the resulting calibration function for a local soil to enhance the sensor’s integration into smart farming solutions, and (iii) investigate the consistency (variation) of the sensor.

## 2. Materials and Methods

### 2.1. Capacitive Soil Moisture Sensors

A capacitive soil moisture sensor estimates the water content of the soil by utilizing its dielectric properties. The sensor operates based on the principle that water has a significantly higher dielectric constant (of approximately 80) compared to dry soil (ranging from 3 to 7) and air (around 1) [47]. This difference allows the sensor to estimate the soil’s moisture content by detecting variations in the dielectric properties of the medium surrounding the sensor.

**Figure 1:**
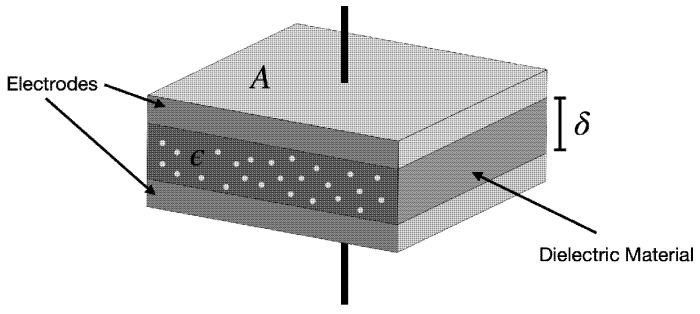
A sketch showing the principle of capacitance soil moisture sensors [48].

A capacitive soil moisture sensor typically consists of two metal plates, or conductive surfaces, that form a capacitor (Figure 1). These plates are placed either inside the soil or in close proximity to it. When an electric field is applied across the plates, the sensor acts as a small capacitor, with the soil representing the dielectric material between the plates.

Capacitance is defined as the ability of a system to store an electrical charge per unit voltage and is expressed as

(1) $$C=\frac{\epsilon A}{\delta }$$

where C is the capacitance in farads (F); ɛ is the dielectric constant (permittivity) of the material, typically expressed as $\epsilon =\epsilon_{0}\epsilon_{r}$, where $\epsilon_{0}$ is the relative permittivity of the material (soil, in this case), which is dimensionless, and ${ɛ}_{r}$ is the vacuum permittivity, a constant value of 8.854 × 10^−12^ (F/m); A is the metal surfaces area (m^2^); and δ is the distance between metallic surfaces (m).

The capacitive soil moisture sensor can be paired with a timer circuit (such as the TLC555 in the case of the SEN0193 sensor) and can output a duty cycle that corresponds to an analog voltage.

As mentioned, in this study, SKU:SEN0193 version 1.2 capacitive soil moisture sensors were used. The sensors are coated with corrosion-resistant materials, have dimensions of approximately 98 mm × 23 mm × 3 mm (length × width × thickness), and weigh around 15 g. They feature an onboard voltage regulator, allowing them to operate within a voltage range of 3.3 to 5.5 V. The sensor includes two pins for power (5 V and Ground) and an analog output pin, making it ideal for interfacing with low-voltage microcontrollers. The output is provided as a frequency signal, ranging from 260 Hz at high moisture levels to 680 Hz at low moisture levels, with higher frequencies indicating lower moisture content [49]. Using the built-in frequency-to-voltage converter circuitry on the sensor, the frequency signal is converted into an output voltage that can be read as an input by the MCU GPIO. The ESP32 MCU has a 12-bit analog-to-digital converter (ADC), which means it can represent this input voltage as an analog value between 0 and 4095.

**Figure 2:**
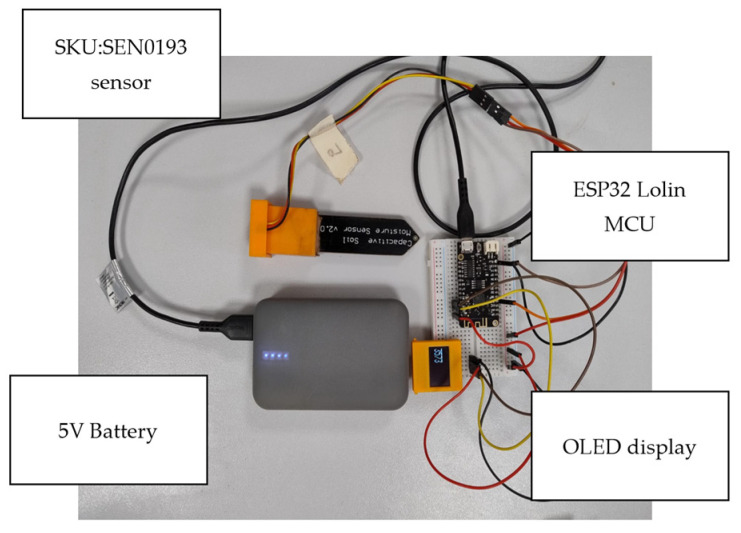
The setup of the soil moisture sensing system.

**Figure 3:**
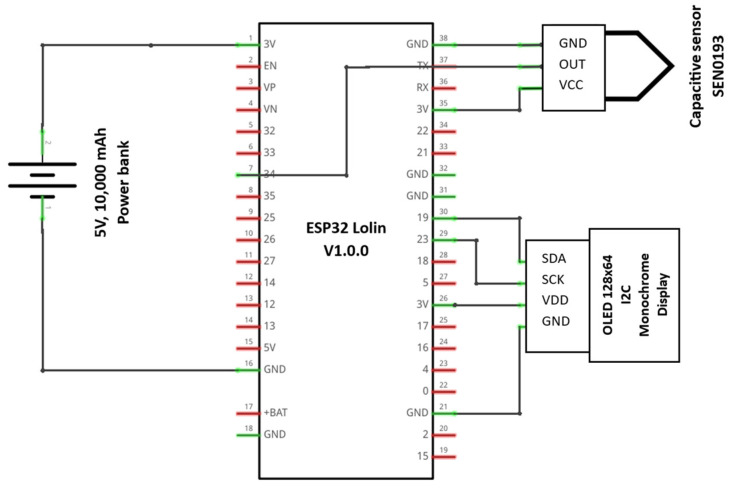
The schematic of the data acquisition system produced by Fritzing.

**Figure 4:**
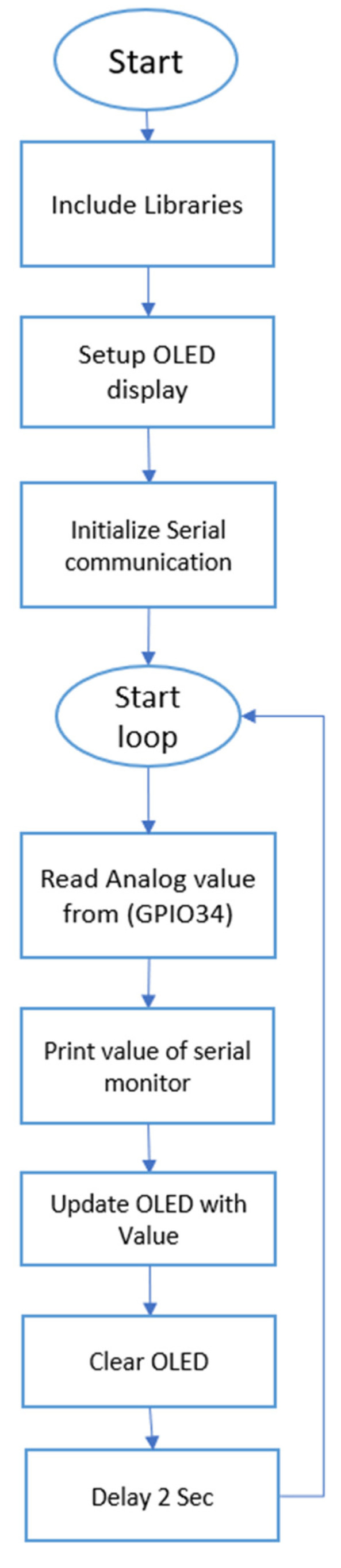
The algorithm flow chart.

The sensor was integrated with the MCU by connecting its output to GPIO pin 34, configured as an analog input. The measured analog signal was processed by the ESP32 and subsequently displayed on the 128 × 64 pixel OLED display interfaced via an I2C protocol, utilizing the dedicated SDA and SCL pins of the microcontroller, GPIO 19 and 23, respectively. The system was programmed to acquire analog readings from the sensor every 2 s, ensuring regular monitoring of soil moisture levels. The processed data were dynamically updated on the OLED display, providing a real-time visualization of the sensor output in a user-friendly manner. Figure 2 shows the setup for the sensor and the microcontroller, while Figure 3 shows the schematic of the connections. The algorithm was written using the C++ programming language. The algorithm flowchart is shown in Figure 4 and was uploaded to the MCU using the Arduino integrated development environment (IDE).

### 2.2. Soil Sampling and the Calibration Process

For the sensors’ calibration, soil samples were collected using an auger from the core soil 10 cm below the soil surface in the experimental field of CIHEAM Bari—Italy, latitude 41°2′40.3872″ N, longitude 16°53′3.8364″ E. The soil is pedologically classified as Colluvic Regosol with a silty loam texture, which consists of 23.5% sand, 59.27% loam, and 17.25% clay; and EC = 0.3 ds/m. It is a soil typical of the Puglia region in southern Italy, representing 20–25% of soil textures in the region and found throughout much of the limestone and calcareous areas [50].

Using the thermogravimetric method [51], a suitable weight (2 kg) of disturbed soil was dried in the oven for 24 h at 105 °C. The average bulk density was 1.1 g/cm^3^. After drying, 15 samples of a known weight (165 g) were prepared in 282 cm^3^ containers. It is important to note that the volume of the sample had to be suitable enough to surround the sensor from all sides as a cylinder, in order to ensure maximum contact. For each set of three sample replicas, the water content was raised by a known amount representing a precalculated percentage of the dried soil weight. The raised steps were 5%, 10%, 20%, 30%, and 40% of gravimetric water content (GWC) (Equation (2)). Water density was assumed to be 1 g/mL (8.25 g or mL of water corresponds to 5% of dry weight):

(2) $$GWC=\frac{m_{w}-m_{d}}{m_{d}}\times 100$$

where $m_{w}$ is the soil wet weight (g), and $m_{d}$ is the soil dry weight (g).

To ensure the homogeneity of water distribution throughout the sample, the soil was mixed after each added dose of water and layered carefully in the container to maximize contact with the sensor.

**Figure 5:**
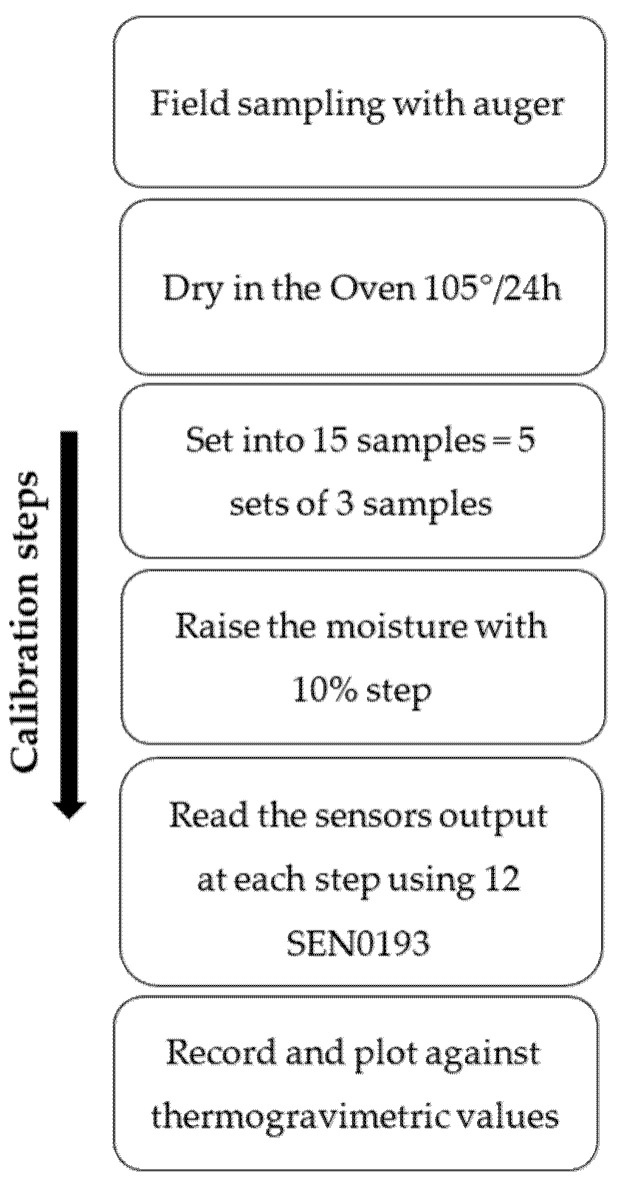
Methodology framework.

**Figure 6:**
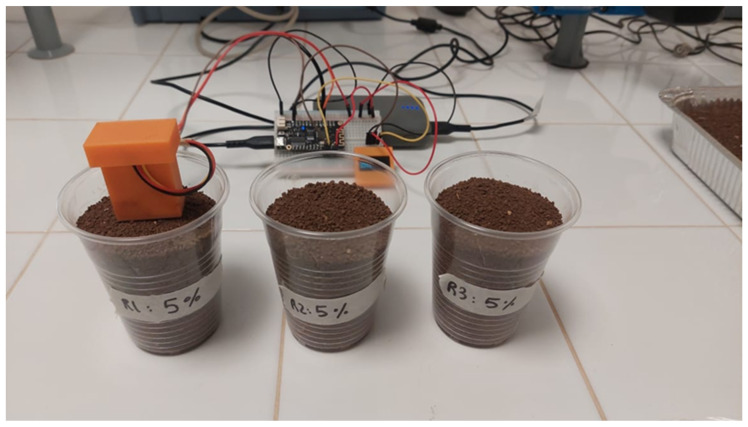
One example of the calibration setup at 5% GWC.

With each raised step of water content, 12 sensors were inserted vertically, and the output voltage of each sensor was read through the ESP32 MCU as an analog value displayed on the OLED screen. A 3D Polyethylene terephthalate glycol (PETG) case was designed and printed to protect the upper part of the sensor where the electronic parts are located. The methodology framework is presented in Figure 5, while Figure 6 shows an example of the calibration setup.

The 180 readings resulting from the five water content steps, three replicas per step, and 12 sensor readings per replica were plotted against the known values of the thermogravimetric water contents. The calibration function was drawn from the linear regression of the resulting curve. Once the calibration function was generalized for the 12 sensors, the accuracy of the sensor was estimated using the coefficient of determination (R^2^) and the root mean square error (RMSE) (Equation (3)). Finally, the coefficient of variation (CV) (Equation (4)) was used to assess the sensor-to-sensor variability in the random sample:

(3) $$RMSE=\sqrt{\frac{\sum_{i=1}^{N}\left\|y\left(i\right)-\bar{y}\left.\left(i\right)\right\|\right.}{N}}$$

where N is the number of data points, $y\left(i\right)$ is the ith measurement, and $\bar{y}(i)$ is its corresponding prediction:

(4) $$CV(\%)=\frac{S}{\bar{X}}\;\times \;100$$

where S is the standard deviation, and $\bar{X}$ is the sample mean.

## 3. Results and Discussion

### 3.1. Sensor Calibration Equation

**Figure 7:**
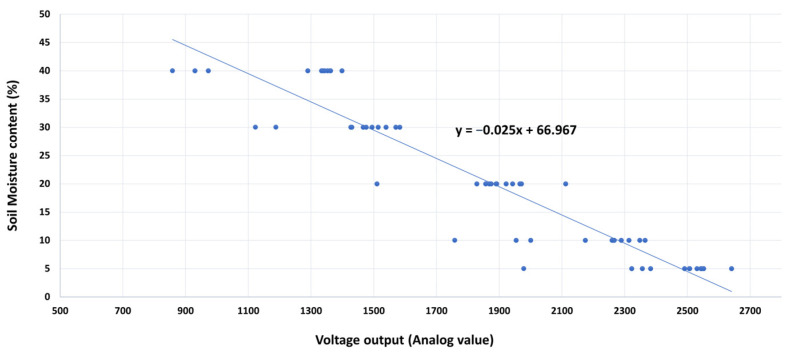
The calibration function for the soil moisture sensor, a = −0.025 and b = 66.967.

Table 1 reports the resulting data set from the sensors’ outputs, while Figure 7 shows the results against the thermogravimetric water content within the investigated soil moisture spectrum. The constant parameters resulting from the linear regression function were used as the guiding calibration function to sense the soil moisture content in our typical soil (silty loam).

### 3.2. Sensor Accuracy

**Figure 8:**
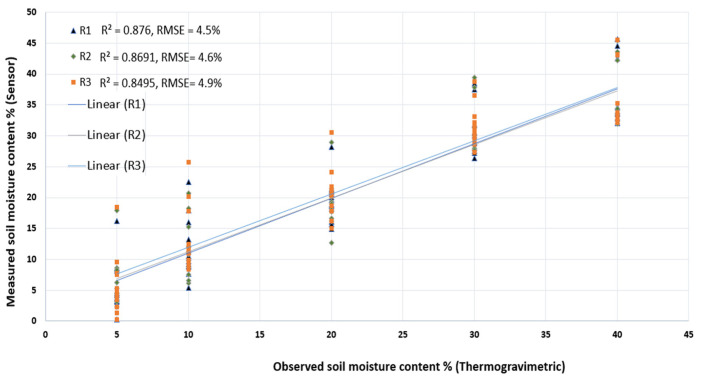
The accuracy of the soil moisture content estimated by the sensor in the three replicas.

Using the resulting calibration function for the typical silty loam soil of the Puglia region, the sensor was able to predict the soil moisture content with relatively high accuracy, where R^2^ ranged between 0.85 and 0.87 for the three replicas, while the RMSE was between 4.5–4.9%, as shown in Figure 8. These results indicate that the calibrated sensor is capable of detecting soil moisture content in this soil with acceptable accuracy and could be integrated into a smart irrigation management system solution. This tailored calibration demonstrates that capacitive sensors, despite their known limitations, can be highly effective in soil moisture monitoring if customized to local soil characteristics, providing a cost-efficient solution for real-time field measurements. However, even if calibrated, real field conditions, such as temperature fluctuations, soil salinity, heterogeneous soil textures, and varying soil compaction, can impact the accuracy of capacitive soil moisture sensors calibrated in controlled laboratory settings. These factors may alter the sensor’s response, leading to deviations from lab-derived calibration curves. Despite these challenges, laboratory calibration remains essential, as it establishes a baseline accuracy and minimizes measurement errors under controlled conditions. By accounting for known variables and creating texture-specific calibration models, lab calibration helps ensure that the sensors perform reliably in diverse field conditions, albeit with potential adjustments needed for site-specific factors.

### 3.3. Sensor Variability and the Coefficient of Variation

**Figure 9:**
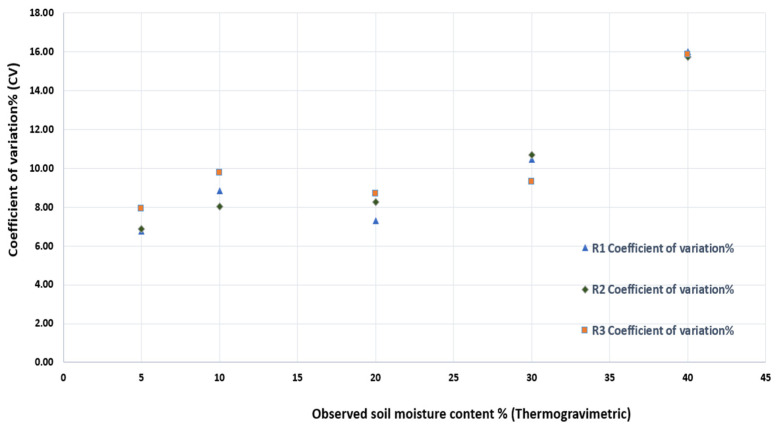
The coefficient of variation between the 12 sensors at each level of soil moisture content for the three replicas.

**Figure 10:**
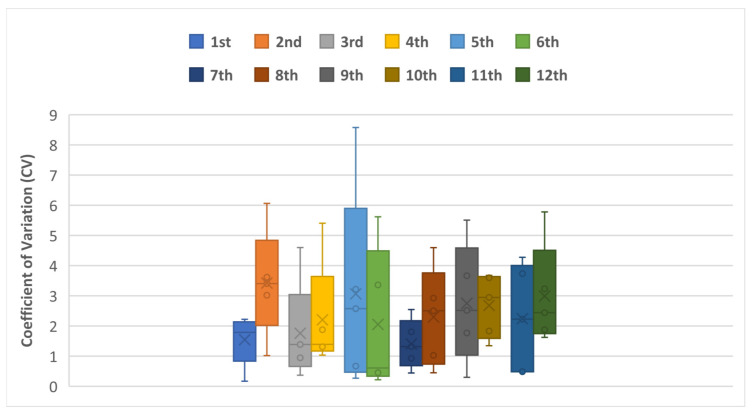
Coefficient of variation of the 12 sensors across the five soil moisture levels.

Another important aspect to investigate is the sensors’ variability. The used generalized calibration function was drawn from a random sample of 12 different sensors. Thus, a more thorough analysis of the sensor-to-sensor variation is important. Figure 9 shows the CV between the 12 sensors for each level of soil moisture content, while Figure 10 shows the variation in each sensor separately.

For the first three soil moisture levels, i.e., 5, 10, and 20%, the sensors showed lower variation, with CV ranging between 6.5 and 10.3%, while with higher soil moisture levels (above 30%), the sensors showed less consistency, and the CV ranged between 10 and 16%. This behavior is mainly due to the working principle of capacitive sensors, as they work by detecting the changes in the dielectric constant of the soil, as previously shown in Equation (1). As mentioned previously, water has a high dielectric constant compared to air and dry soil, so as soil moisture increases, the dielectric constant changes. However, at higher moisture levels, the dielectric constant changes less significantly with additional water, making it harder for the sensor to detect small variations, resulting in lower sensitivity and higher CVs. Table 2 reports the calculated statistical indicators between the 12 sensors at each soil moisture level.

## 4. Conclusions

Advancements in electronic technology have enabled researchers to access affordable solid-state sensors and programmable microcontroller-based circuits. In this study, a low-cost soil moisture sensor was calibrated under laboratory conditions. The calibration equation for the sensor was determined, and results indicate that the SEN0193 capacitive soil moisture sensor, when adjusted for the specific properties of the silty loam soil in the Puglia region in Italy, achieves a high predictive accuracy for soil moisture content. With a coefficient of determination (R^2^) ranging between 0.85 and 0.87 and an RMSE ranging between 4.5 and 4.9% across three replicas, the sensor demonstrated a strong correlation between observed and predicted values, highlighting its effectiveness in capturing moisture variations under these specific soil conditions. This result suggests a high degree of reliability in the sensor’s readings, showing that it is well-suited for smart irrigation management applications.

This study’s findings enhance the integration of SEN0193 capacitive soil moisture sensors by providing a tailored calibration function that could be used for silty loam soils. The findings underscore the impact of region-specific calibration for capacitive sensors, which tend to have higher error margins in generalized applications due to soil textures and salinity. By calibrating the sensor, this study reduced such errors, thereby enhancing the sensor’s integrability for precision agricultural practices in similar semi-arid, silty loam soils.

This research adds to the growing body of knowledge regarding low-cost precision agriculture tools, suggesting that capacitive sensors are a viable and cost-effective alternative to more complex methods for soil moisture monitoring that could be easily integrated into farming systems. However, the impact of such integration should be quantified in terms of water productivity and water savings compared to conventional irrigation management systems, which will be a subject for future studies.

## Figures and Tables

**Table 1:** The output analog readings from the 12 sensors under all moisture levels.

Probe No.	Gravimetric Water Content, %	Voltage (Analog Output)			
		Rep. 1	Rep. 2	Rep. 3	Average
1	5	2509	2544	2469	2507.33
	10	2320	2238	2241	2266.33
	20	1922	1845	1856	1874.33
	30	1407	1419	1456	1427.33
	40	1339	1343	1343	1341.67
2	5	2336	2336	2295	2322.33
	10	1963	2070	1968	2000.33
	20	1953	1905	1818	1892.00
	30	1524	1567	1393	1494.67
	40	895	937	957	929.67
3	5	2497	2465	2511	2491.00
	10	2271	2227	2293	2263.67
	20	1857	1821	1808	1828.67
	30	1462	1473	1354	1429.67
	40	1330	1338	1339	1335.67
4	5	2340	2429	2378	2382.33
	10	2150	2194	2180	2174.67
	20	1827	1969	2032	1942.67
	30	1410	1424	1447	1427.00
	40	1296	1303	1269	1289.33
5	5	2525	2495	2497	2505.67
	10	2291	2434	2320	2348.33
	20	2033	1858	1713	1868.00
	30	1477	1497	1424	1466.00
	40	1339	1344	1346	1343.00
6	5	2549	2554	2525	2542.67
	10	2325	2201	2342	2289.33
	20	2053	2015	1845	1971.00
	30	1478	1472	1477	1475.67
	40	1339	1334	1327	1333.33
7	5	2535	2518	2539	2530.67
	10	2257	2244	2285	2262.00
	20	1895	1833	1842	1856.67
	30	1521	1561	1535	1539.00
	40	1321	1376	1385	1360.67
8	5	2530	2502	2624	2552.00
	10	2371	2379	2191	2313.67
	20	1931	2032	1934	1965.67
	30	1589	1575	1584	1582.67
	40	1362	1376	1348	1362.00
9	5	2667	2587	2669	2641.00
	10	2462	2416	2217	2365.00
	20	2083	2173	2079	2111.67
	30	1626	1574	1511	1570.33
	40	1397	1403	1395	1398.33
10	5	2554	2495	2587	2545.33
	10	2309	2286	2184	2259.67
	20	1876	1919	1874	1889.67
	30	1575	1471	1495	1513.67
	40	1295	1384	1378	1352.33
11	5	2353	2347	2369	2356.33
	10	2039	1951	1872	1954.00
	20	1839	1958	1968	1921.67
	30	1178	1168	1218	1188.00
	40	855	863	857	858.33
12	5	2032	1963	1939	1978.00
	10	1776	1850	1649	1758.33
	20	1551	1523	1456	1510.00
	30	1139	1103	1126	1122.67
	40	976	989	953	972.67

**Table 2:** The calculated statistical indicators for the resulting dataset.

	SWC	Rep. 1	Rep. 2	Rep. 3
Mean	5	2452.25	2436.25	2450.17
	10	2211.17	2207.50	2145.17
	20	1901.67	1904.25	1852.08
	30	1448.83	1442.00	1418.33
	40	1228.67	1249.17	1241.42
Standard Deviation	5	165.64	167.60	193.94
	10	195.22	177.28	209.34
	20	138.86	156.99	160.68
	30	151.43	154.18	131.98
	40	196.60	196.34	196.55
Coefficient of variation	5	6.75	6.88	7.92
	10	8.83	8.03	9.76
	20	7.30	8.24	8.68
	30	10.45	10.69	9.31
	40	16.00	15.72	15.83

## Data Availability

Data are contained within the article.
